# The Effects of Physical Inactivity and Exercise at Home in Young Patients with Congenital Heart Disease during the COVID-19 Pandemic

**DOI:** 10.3390/ijerph181910065

**Published:** 2021-09-25

**Authors:** Federica Gentili, Giulia Cafiero, Marco Alfonso Perrone, Massimiliano Bianco, Annamaria Salvati, Ugo Giordano, Stefani Silva Kikina, Paolo Guccione, Andrea De Zorzi, Lorenzo Galletti, Fabrizio Drago, Benedetta Leonardi, Attilio Turchetta

**Affiliations:** 1 Division of Sports Medicine, Bambino Gesù Children’s Hospital IRCCS, 00165 Rome, Italy; federica.gentili@opbg.net (F.G.); giulia.cafiero@opbg.net (G.C.); drsalvati@yahoo.it (A.S.); ugo.giordano@opbg.net (U.G.); attilio.turchetta@opbg.net (A.T.); 2 Department of Cardiology and Cardiac Surgery, Bambino Gesù Children’s Hospital IRCCS, 00165 Rome, Italy; stefanisilva.kikina01@icatt.it (S.S.K.); paolo.guccione@uniroma2.it (P.G.); andrea.dezorzi@opbg.net (A.D.Z.); lorenzo.galletti@opbg.net (L.G.); fabrizio.drago@opbg.net (F.D.); benedetta.leonardi@opbg.net (B.L.); 3 Department of Cardiology and University Sports Centre, University of Rome Tor Vergata, 00133 Rome, Italy; 4 Unità Operativa Complessa di Medicina dello Sport e Rieducazione Funzionale, Fondazione Policlinico Universitario Agostino Gemelli IRCCS, Università Cattolica del Sacro Cuore, 00168 Rome, Italy; massimiliano.bianco@policlinicogemelli.it

**Keywords:** congenital heart disease, COVID-19, exercise training, physical activity

## Abstract

**Background:** The COVID-19 pandemic had a significant impact on the population’s ability to be physically active. **Purpose:** Evaluate the effect of the COVID-19 mitigation measures on exercise tolerance in patients with congenital heart disease (CHD). **Materials and methods:** All subjects (880, 6–18 years old) who performed a stress test at our hospital from October 2020 to February 2021 and had a similar test one year earlier were enrolled. A questionnaire on the degree of physical activity carried out in 2020 concerning the period prior to the pandemic was compiled. Exercise tolerance and the main anthropometric parameters between the first and second tests were compared. **Results:** 110 subjects (11.9 ± 4.1 years) were included in the study. The percentage of patients engaged in regular physical activity (RPA) decreased significantly during the pandemic (*p* < 0.001), and BMI increased significantly (*p* < 0.001), except among the subjects who began RPA during the lockdown, whereas test duration did not decrease significantly overall but increased in this last subgroup (*p* < 0.05) **Conclusions:** The COVID-19 lockdown led to a less active lifestyle with a significant increase in BMI in our group of CHD. These data could have negative effects on the risk profile of this population. RPA practiced at home seems to be effective in counteracting such effects.

## 1. Background

Coronavirus disease 2019 (COVID-19) is an ongoing pandemic caused by the severe acute respiratory syndrome coronavirus 2 (SARS-CoV-2), which has infected nearly 200 million people worldwide so far [1,2,3].

On 30 January 2020, the World Health Organization (WHO) declared the COVID-19 epidemic a global health emergency, and on 11 March 2020, the disease was declared a global pandemic [1]. The consequences in terms of human lives and hospital admissions have been dramatic [2,3]. In fact, to date, more than 4 million people have died in the world from the coronavirus outbreak [2].

In order to limit the spread of the virus and the saturation of intensive care units, measures have been taken to confine individuals to their homes and to promote social distancing. As a result, gyms, public parks, sports fields, outdoor play areas, and schools, as well as multiple commercial activities, have been closed. This has significant consequences on the possibility of practicing physical activity (PA) and sports in the general population, in particular children and young people [4,5,6].

As previously defined [7,8], PA is any bodily movement produced by skeletal muscles that results in energy expenditure, whereas exercise or exercise training is a structured and regular physical activity (RPA) that is purposeful to improve or maintain one or more components of physical fitness. It is widely known that RPA reduces the risk of many adverse health outcomes, having an inverse relationship with early mortality, cardiovascular diseases, hypertension, stroke, osteoporosis, type 2 diabetes, metabolic syndrome, obesity, cancers, and depression [8,9,10]. There is a dose–effect relationship between RPA and cardiovascular (CV) or all-cause mortality, with a 20–30% reduction in adverse events compared with sedentariness [11,12]. Several studies have shown that RPA correlates with a reduction in CV adverse events in the general population and especially in selected patient populations, such as patients with congenital heart disease [7,8,9,10,11,12]. In fact, people with congenital heart disease (CHD) are among those for whom it is particularly important to maintain an active and healthy lifestyle [7,8]. Recent studies have shown that, in patients with CHD, RPA is associated with improved cardiorespiratory and musculoskeletal performance, a reduction in heart failure biomarkers, a better quality of life, and higher levels of self-esteem [13,14,15,16,17,18,19,20]. It has also been noted that RPA contributes to reducing obesity and other CV risk factors, whose prevalence is particularly high in both children and adults with CHD [21,22,23]. Therefore, exercise performed following a well-defined methodology [7] can be considered real therapy for these patients. Moreover, the effects of physical inactivity due to the COVID-19 lockdown in children and adolescents with CHD are still poorly studied. Therefore, the purpose of this retrospective study was to evaluate the effects of reduced PA and exercise training at home during the COVID-19 pandemic on the exercise tolerance and body mass index (BMI) of children and adolescents with CHD.

## 2. Methods

This cohort study had a retrospective observational design. Patients with CHD were regularly followed at our day hospital and monitored every six to twelve months. The study was approved by the Ethics Committee of our Institution (Prot. number 982 OPBG 2015). All subjects signed an informed consent form. The study was conducted in accordance with the current version of the Declaration of Helsinki (2013).

All young patients who underwent stress tests at the Department of Pediatric Cardiology and Cardiac Surgery of Bambino Gesù Children’s Hospital IRCCS in the period from October 2020 to February 2021 were evaluated retrospectively.

The inclusion criteria were

– good hemodynamic compensation (NYHA 1 or 2), without moderate/severe ventricular dysfunction and/or history of significant arrhythmias requiring interventional therapies or procedures. These patients regularly receive a prescription for RPA practice during their evaluation in our institution, for which parental supervision is usually not considered necessary, although recommended, due to their low-risk profile;
– availability of a previous exercise test carried out from October 2019 to February 2020.

Exclusion criteria were

– presence of significant clinical and/or therapeutic changes between the two tests, which made the tests non-comparable;
– history of COVID-19 infection;
– early interruption of one of the tests for reasons other than muscle exhaustion, the perception of a maximal effort (perceived fatigue of 8 on a scale from 1 to 10), and/or the achievement of a maximal heart rate (HR) (>85% of theoretical maximal HR based on individual age in patients without beta-blocker therapy).

Before the stress test, all subjects were routinely questioned on their degree of PA carried out in the period prior to and during the pandemic. A single operator (G.F.) investigated all subjects for the type, frequency, duration, and intensity of PA practiced in 2020 with regard to the period preceding the spread of the pandemic and during the confinement period. We considered as engaged in RPA the subjects who met the current 2020 ESC guidelines [8] of a minimum of 150 min of moderate-intensity (rate of perceived exertion 12–13 on the Borg 6–20 scale) endurance exercise training over 5 days or 75 min of vigorous (rate of perceived exertion 14–16 on the Borg 6–20 scale) exercise per week over 3 days. Regardless of the practice of RPA, all subjects were also questioned about their perceived lifestyle based on the hours of sedentary time spent daily.

All exercise tests examined were carried out on a treadmill using the Bruce protocol, with continuous 12-lead electrocardiographic monitoring, periodic measurement of blood pressure (BP), and pulse oximetry.

The parameters detected during each test were age, gender, weight (kg), height (m), BMI, test duration (s), heart rate (HR) at the moment of peak exercise in absolute value and as a percentage of the theoretical maximum HR (defined according to Cooper’s Equation as 220-age), and the days elapsed between the start of the restriction measures and the second test.

## 3. Statistics

Jamovi V.1.6 was used for the analysis [24]. Continuous variables were expressed as means and standard deviations (normality was checked by the Shapiro–Wilk test), while the categorical variables were summarized as counts and percentages. The degree of effort tolerance (indirectly defined by the test duration) and the main anthropometric parameters between the first and second tests were compared using Student’s t-test for paired data. Categorical variables were compared by chi-square or Fisher’s exact test, when appropriate. The correlation between the days elapsed between the start of the restrictions and the second test and effort tolerance or main anthropometric parameters was checked by means of Pearson’s correlation coefficient. Finally, the duration of the treadmill tests was compared with the median value reported in the literature for healthy subjects of the same age, and differences between genders or types of disease were investigated. In particular, based on what has been reported in the medical literature by van der Cammen [25], subjects were divided into tertiles, with the first tertile having the lower exercise tolerance (only the subjects with age falling in the age groups analyzed in van der Cammen’s paper were included in the analysis). The tertiles were compared using the Tukey post-hoc test, and test durations (pre-pandemic vs. post-mitigation measures) of all single tertiles were compared using Student’s t-test for paired data.

Differences were considered statistically significant when *p* ≤ 0.05.

## 4. Results

A total of 880 individuals (age 6–18 years) underwent a stress test at our department during the study period (October 2020 to February 2021). Of them, 110 subjects (53 females) met the inclusion criteria. The main characteristics of this group are summarized in Table 1. The percentage of patients engaged in RPA decreased significantly in the period of the pandemic compared to the previous period (61.8% vs. 12.7%; *p* < 0.001). Regardless of RPA practice, 93 subjects (84.5%) reported a deterioration of their lifestyle in terms of increased sedentary time.

Significant increases in stature (1.49 ± 0.17 m vs. 1.53 ± 0.16 m, *p* < 0.001) and weight (44.6 ± 17.2 kg vs. 50.5 ± 18.1 kg, *p* < 0.001) were observed between the first and second tests. Interestingly, BMI was significantly higher in the second test than in the first one (19.35 ± 4.33 kg/m^2^ vs. 21.0 ± 4.61, *p* < 0.001), although the percentage of obese (BMI ≥30 kg/m^2^) and overweight (BMI ≥25 kg/m^2^) subjects did not change significantly (1.8% of obese subjects in both the first and second tests and 11.8% of overweight subjects in the first test vs. 17.3% in the second test). A statistically significant correlation emerged (r = 0.173, *p* = 0.035) between BMI and the number of days elapsed between the start of the restrictions and the second test. The significant increase in BMI emerged in comparing the tests of 14 subjects who reported being physically active before and during the pandemic (*p* < 0.001), as well as in comparing the tests of 61 subjects who suspended RPA during the pandemic (*p* < 0.001) or who were already previously inactive (n = 35 individuals, *p* < 0.001). On the contrary, we did not find a significant increase in BMI among the subjects (n = 7) who were previously inactive and began RPA at home during the lockdown (*p* = 0.137).

The duration of the test did not change significantly between the two exams in the whole population (mean duration 595 ± 118 s vs. 603 ± 120 s) and in the different subgroups, except for the subgroup of subjects who began RPA at home during the lockdown (Table 2), in which a significant increase in the test duration was observed (*p* < 0.05).

When comparing our results on the duration of the first treadmill test with what was reported in the literature on healthy subjects of the same age, only 20.5% of our patients had a test duration equal to or higher than the median value reported by van der Cammen et al. [25], and no significant difference was observed between genders (580 ± 96 s in girls, 587 ± 126 in boys). Overall, our patients had a lower test duration (mean value −140 ± 116 s) than expected. Comparisons among tertiles regarding the treadmill test duration are shown in Table 3. Looking at different diagnoses in the first and third tertiles, we observed a higher rate of patients who underwent the Fontan procedure (29.6%) in the first and a higher rate of patients with corrected Fallot (29.6%) in the third. However, these differences did not reach statistical significance. No other factor usually adopted to stratify the risk in patients with CHD (i.e., NYHA class, left ventricular dysfunction, right ventricular dysfunction, pulmonary hypertension, oxygen desaturation during the stress test, frequent and/or complex arrhythmias) had a different rate when we compared these two subgroups.

## 5. Discussion

The social distancing measures implemented to limit the spread of the COVID-19 pandemic have profoundly changed the population’s lifestyle, reducing their ability to practice PA and leading to a higher degree of sedentary behavior, as documented by several recent studies [26,27,28,29,30,31,32,33]. A retrospective study on Spanish students enrolled in 16 universities, involving a total of 13 754 valid survey responses, described reduced moderate (−29.5%) and vigorous (−18.3%) PA during confinement and increased sedentary time (+52.7%) [29]. Several observational cohort studies have reported comparable declines in the degree of PA among adolescents [30,31,32,33]. The study analysis designed by Ammar et al. [26] including 1047 reports (54% women) from Asia, Africa, Europe, and other countries, derived from an electronic survey (ECLB-COVID19- electronic survey), documented a negative effect on all levels of PA intensity, an increase in daily sitting time (5–8 h per day), and a worsening of eating habits (type of food, out of control eating, snacking between meals, and the number of main meals). The data that emerged from the analysis of our 110 patients (53 females) with CHD seem to agree with these observations, with 49.1% of the subjects who stopped RPA during the pandemic and 84.5% of the subjects who reported lifestyle deterioration with a higher level of sedentary time.

Contrary to what was expected and documented in other studies [30,31,32,33], no significant worsening in exercise tolerance was observed in our group of young subjects with CHD. By using the Bruce protocol, stress tolerance can be indirectly derived from the exercise duration when the same individual is retested, particularly when the time between the two tests is quite short, as in our study. The exercise duration in our patients turned out not to be significantly different between the two tests (mean duration 595 ± 118 s at the first vs. 603 ± 120 s at the second test). However, the potential improvement in coordination skills between the first and second test must be considered in determining these data, both for a “learning” factor and for the different stages of development of the children themselves (average age 11.85 ± 4.14 years vs. 12.83 ± 4.17 years, average height 1.49 ± 0.17 m vs. 1.53 ± 0.16 m). Therefore, we can state that a lack of improvement (that was potentially expected) in exercise capacity on the treadmill tests of these patients was observed. In particular, data from healthy children show a proportional increase in treadmill test duration (Bruce protocol) until the age of 12–13 in boys, while girls tend to show a decline after peaking at 10–11 years [25,34]. Assuming that the expected increase in the duration of exercise testing fluctuates between 0.2 and 0.8 min/year for boys and −0.4 and −0.1 min/year for girls at the age of 11–12 years [25,34], then the apparent lack of change in the test duration in our growing pediatric population with CHD could hide a worsening physical performance. Unfortunately, we cannot draw definitive conclusions on this aspect, since no reference data have been published yet on a pediatric population with CHD. Moreover, our study design did not allow us to evaluate the effect of CHD and COVID-19 mitigation measures on the duration of treadmill testing, as we did not have a control group of healthy subjects of the same age analyzed in the same period of time.

Looking in detail at our study group, when the first treadmill test was analyzed, we observed that only 20.5% of our patients had a test duration equal to or higher than the median value reported by van der Cammen et al. for healthy subjects of the same age [25], with a mean test duration lower than expected (−140 ± 116 s). Looking for possible explanations on factors that could have influenced this aspect, we compared the tertiles of our sample based on the expected test duration of each child [25]. As predicted, there was a significant difference in treadmill test duration among tertiles (Table 3), both in the first and second tests. However, even though we had a higher rate of patients who underwent the Fontan procedure (29.6%) in the first tertile and a higher rate of patients with corrected Fallot (29.6%) in the third, these differences were not significant. Furthermore, no other factor usually adopted to stratify the risk in patients with CHD (i.e., NYHA class, left ventricular dysfunction, right ventricular dysfunction, pulmonary hypertension, oxygen desaturation during the stress test, frequent and/or complex arrhythmias) had a different rate when we compared the tertiles. No significant difference was found between the two treadmill tests (pre-pandemic and post-mitigation measures) in each tertile.

When anthropometric characteristics were analyzed, a significant increase in body weight was observed in the whole population, both in absolute value (44.6 ± 17.2 kg vs. 50.5 ± 18.1 kg, *p* < 0.001, as partly expected for the different evolutionary phase of observation) and in terms of BMI (19.35 ± 4.33 kg/m^2^ vs. 21.0 ± 4.61 kg/m^2^, *p* < 0.001, an index commonly used to assess the degree of body fatness). Unfortunately, we did not evaluate the body composition of our subjects. This aspect could be very useful in understanding whether the increase in BMI was due to an increase in fat-free mass, fat mass, or both and how these variables could have influenced exercise tolerance. Interestingly, a statistically significant correlation emerged (r = 0.173, *p* = 0.035) between the BMI at the second test and the number of days elapsed between the start of the restrictions and the second test itself, an index of the length of the period spent with a reduced level of PA. Similar results were observed by Pietrobelli et al., analyzing the effects of COVID-19 restrictions on lifestyle behaviors of obese children in Italy. The main findings from this study similarly showed that the participants were less active (time spent in sports activities decreased by 2.30 ± 4.60 h/week) during the COVID-19 home confinement period and had consequently gained weight due to this unhealthy lifestyle [35]. In addition, previous studies revealed that the school environment reduces obesity risks because it provides routine and structure in daily life [36].

This significant increase in BMI among our patients emerged in comparing the tests of subjects who were engaged in RPA both before and during the pandemic (*p* < 0.001), as well as in comparing the tests of subjects who suspended RPA during the pandemic (*p* < 0.001) or who already had been previously inactive (*p* < 0.001). Instead, we did not find a significant increase in BMI in the seven subjects who were previously inactive and voluntarily began RPA at home during the lockdown (*p* = 0.137). Moreover, a significant increase in exercise tolerance was observed (*p* < 0.05) only in this subgroup of patients. In this regard, other authors have also reported the need for home exercise training to maintain an active lifestyle during home confinement [37], suggesting the use of suitable telemedicine programs [35] and providing specific indications on exercise training to be carried out at home [38]. The prescription of RPA must be considered as a real therapeutic approach to cardiovascular diseases. Furthermore, it is important to raise patient awareness on the importance of not interrupting PA and to encourage them to continue PA by providing them with programs that can also be applied at home. In this regard, many studies have demonstrated the feasibility, safety, and efficacy of different models of home-based RPA [35,36,37,38,39]. Patients with better cardiorespiratory fitness are less vulnerable to viruses and, therefore, maintaining RPA is crucial during the pandemic [40]. Caution should be exercised when prescribing home-based RPA for high-risk patients, however, who were not present in our study. For those, closer remote supervision using telecommunication might be necessary [38,39,40,41].

Technological advancements over recent years have boosted the emergence of a multitude of tools, such as PA trackers and applications for smartwatches and phones, that can help to monitor PA interventions [38,39]. In fact, physical exercise prescription protocols and continuous monitoring are necessary for the management of this population. Therefore, it is important to follow specific indications in terms of intensity, frequency, volume, and mode of exercise in the planning and carrying out RPA in patients with CHD, to be adapted to each individual patient [7,8,38,42,43,44].

## 6. Limitations of The Study

The observation period considered includes periods of total lockdown, with confinement at home and periods with less important limitations associated with the resumption (although in many cases discontinuous) of face-to-face teaching for primary school subjects and the temporary reopening of some sports activities. This aspect could limit the homogeneity of the acquired data. However, this is a global look at the effects of the lifestyle changes in the population in 2020 due to the measures that were necessary to limit the spread of the pandemic.

Another limitation of our study is that we enrolled subjects with different CHD that may have interfered differently with their exercise capacity. However, our aim was not to compare subjects with different CHD but to verify, in the same subject (with the same CHD), the change in exercise tolerance someway related to the mitigation measures imposed by the COVID-19 pandemic.

## 7. Conclusions

The closures related to the COVID-19 mitigation measures have led to a less-active lifestyle with a significant increase in BMI in our group of pediatric subjects with CHD, which significantly correlates with the number of days elapsed since the beginning of the closures. While not indicating a clear reduction in exercise tolerance, these data could have particularly adverse effects on the risk profile of this particular population with CHD. Home-based regular physical activity seems to be an effective means of reducing/offsetting the negative effects caused by such measures and should be considered in similar situations in the future.

## Figures and Tables

**Table 1:** Main characteristics and type of pathology in 110 young patients with congenital heart disease who composed the study population. * = *p* < 0.05.

Main Characteristics	First Test	Second Test
Age at Test (Years) (Mean; SD)	11.85 (4.14)	12.83 (4.17) *
Physically Active Subjects (Number; %)	68 (61.8)	14 (12.7) *
Weight (kg) (Mean; SD)	44.59 (17.20)	50.50 (18.08) *
Height (m) (Mean; SD)	1.49 (0.17)	1.53 (0.16) *
BMI (Mean; SD)	19.35 (4.33)	20.99 (4.61) *
**Types of Congenital Heart Disease**	**Number of Subjects (%)**	
○ Post-Fontan Intervention	25 (22.7%)	
○ Corrected TOF	20 (18.2%)	
○ Corrected COA, AA Anomalies (Corrected DAA and Aortic Kinking)	16 (14.5%)	
○ TGA Post-Arterial Switch Operation	15 (13.6%)	
○ Aortic Valve Disease in Natural History	10 (9.1%)	
○ Corrected CAVC	8 (7.3%)	
○ CA (Post-KD, Abnormal Coronary Origin)	6 (5.5%)	
○ Ebstein’s Anomaly	5 (4.5%)	
○ Corrected TAPVR or PAPVR	3 (2.7%)	
○ Post-Ross Intervention	1 (0.9%)	
○ Corrected Aortopulmonary Window	1 (0.9%)	
NYHA Class I	108 (98.2%)	
NYHA Class II	2 (1.8%)	
Left Ventricular Dysfunction (Mild)	4 (3.6%)	
Right Ventricular Dysfunction (Mild)	4 (3.6%)	
Pulmonary Hypertension	9 (8.2%)	
Frequent and/or Complex Arrhythmias During the Stress Test	0 (0%)	
Oxygen Desaturation During the Stress Test (≤90%)	0 (0%)	

Legend: TOF = Tetralogy of Fallot; TGA = Transposition of Great Arteries; COA = Aortic Coarctation; AA = Aortic Arch; DAA = Double Aortic Arch; CAVC = Complete Atrioventricular Canal; CA = Coronary Abnormalities; KD = Kawasaki Disease; TAPVR = Total Pulmonary Venous Return; PAPVR = Partial Anomalous Pulmonary Venous Return.

**Table 2:** Comparison of treadmill test duration (s) between the first test held before the pandemic (pre) and the second test held during the pandemic (post) in a group of young patients with congenital heart disease. RPA: regular physical activity.

	Number of Subjects	Test Duration Pre (s)	Test Duration Post (s)	*p*-Value
**RPA Pre and Post**	7	686 ± 167	690 ± 149	0.257
**RPA Only Pre**	61	617 ± 118	611 ± 125	0.197
**RPA Only Post**	7	520 ± 93	604 ± 94	< 0.05
**No RPA**	35	553 ± 88	571 ± 103	0.398
**Total**	110	595 ± 118	603 ± 120	0.306

**Table 3:** Bruce protocol test duration in a group of young subjects with congenital heart disease compared to what has been reported in the literature for healthy subjects of the same age **(25)**. The overall group was divided into tertiles based on the expected test duration (the first tertile has the lowest exercise tolerance).

	Test Duration Pre (s)	Test Duration Post (s)	Difference in Respect to Expected Values Pre (s)	Difference in Respect to Expected Values Post (s)
**First Tertile**	488 ± 81 ^&	519 ± 106 &	−273 ± 72 ^&	−242 ± 98 ^&
**Second Tertile**	574 ± 56 &	569 ± 79 &	−126 ± 22 &	−130 ± 77 &
**Third Tertile**	584 ±.112 ^	593 ± 116 ^	−140 ± 116 ^	−131 ± 122 ^

^ = *p* < 0.005 comparing the second tertile with the others at each time point (pre or post); & = *p* < 0.005 comparing the third tertile with the others at each time point (pre or post).
